# The feasibility of early detecting coronary artery disease using deep learning-based algorithm based on electrocardiography

**DOI:** 10.18632/aging.204688

**Published:** 2023-05-01

**Authors:** Panli Tang, Qi Wang, Hua Ouyang, Songran Yang, Ping Hua

**Affiliations:** 1 Department of Cardiovascular Surgery, Sun Yat-sen Memorial Hospital, Sun Yat-sen University, Guangzhou 510120, China; 2 The Biobank of Sun Yat-sen Memorial Hospital, Sun Yat-sen University, Guangzhou 510120, China

**Keywords:** artificial intelligence, deep learning, coronary artery disease, electrocardiography, diagnosis

## Abstract

Background: Coronary Artery Disease (CAD) is a major cause of morbidity and mortality, yet it is frequently asymptomatic in the early stages and hence goes undetected.

Objective: We aimed to develop a novel artificial intelligence-based approach for early detection of CAD patients based solely on electrocardiogram (ECG).

Methods: This study included patients with suspected CAD who had standard 10-s resting 12-lead ECGs and coronary computed tomography angiography (cCTA) results within 4 weeks or less. The ECG and cCTA data from the same patient were matched based on their hospitalization or outpatient ID. All matched data pairs were then randomly divided into training, validation dataset for model development based on convolutional neural network (CNN) and test dataset for model evaluation. The accuracy (Acc), specificity (Spec), sensitivity (Sen), positive predictive value (PPV), negative predictive value (NPV) and area under the receiver operating characteristic curve (AUC) of the model were calculated by using the test dataset.

Results: In the test dataset, the model for detecting CAD achieved an AUC of 0.75 (95% CI, 0.73 to 0.78) with an accuracy of 70.0%. Using the optimal cut-off point, the CAD detection model had sensitivity of 68.7%, specificity of 70.9%, positive predictive value (PPV) of 61.2%, and negative predictive value (NPV) of 77.2%. Our study demonstrates that a well-trained CNN model based solely on ECG could be considered an efficient, low-cost, and noninvasive method of assisting in CAD detection.

## INTRODUCTION

Cardiovascular diseases (CVDs) continue to be the leading cause of death worldwide, accounting for approximately 33% of global mortality in 2019 [1, 2]. In China, nearly 3.5 million people die each year from CVDs or CVD-related diseases, accounting for up to 45.0% of deaths in rural areas and 42.6% in cities [3, 4]. The most common type of CVDs was coronary artery disease (CAD), which is the clinical consequence of atherosclerotic plaque formation, resulting in reduced blood supply to the distal myocardium, i.e., ischemia [5, 6]. Despite this, CAD patients may go years without a diagnosis because there are only minor or non-identifiable symptoms in the early stages [7]. There is a high prevalence of asymptomatic CAD in diabetics, particularly those with type 2 diabetes mellitus [8]. CAD continues to be a substantial cause of poor life quality and increased healthcare expenditure and as a result, there is still an urgent unmet need to develop a new strategy for early detection of CAD, which is a common challenge in the world that the clinicians are currently facing [9, 10].

Traditionally, for patients with suspected CAD, invasive coronary angiography (CAG) or coronary computed tomography angiography (cCTA) has been the preferred method for diagnosis [11]. Routine screening with CAG or cCTA, on the other hand, is not justified for patients with transient episodes of chest pain or mild symptoms due to cost, time consuming, invasive procedural risks, radiation, and potentially nephrotoxic iodinated contrast exposure [12, 13]. The conventional 12-lead electrocardiogram (ECG) is a low-cost, widely used, and non-invasive medical tool used on patients for both cardiac and noncardiac reasons [14]. In the clinical setting, electrophysiologists or cardiologists examine both rhythmic and morphologic ECG abnormalities. However, analyzing a wide range of ECGs from a variety of patients is time-consuming and highly dependent on the expertise of the individual. In addition, in the early stages of cardiac dysfunction, patients typically present mild signs and symptoms or are even asymptomatic, resulting in only minor changes in ECG [15, 16]. Until now, there have been no widely used screening strategies for patients with silent or minimally symptomatic CAD.

Deep learning (DL), which has revolutionized artificial intelligence (AI), is currently the most advanced branch of machine learning (ML) [17, 18]. It has made remarkable progress in a wide range of applications, including computer vision, speech recognition, and natural language processing [19]. DL has a powerful potential for automatically discovering the abstract representations required for prediction from the raw data without substantial information loss [20–23], which has resulted in promising achievements in medical fields, such as disease screening, diagnosis, and prediction. CNNs are one of the most successful DL architectures which have received enormous attention in the fields of medicine in the last 5 years [19]. The CNN application for digital ECG data could detect subtle changes in ECGs that were related to cardiac structural or functional abnormalities. A recent Lancet study, for example, had shown that an AI-enabled ECG could be used for recognition of patients with paroxysmal atrial fibrillation (AF) during sinus rhythm based solely on ECG [15]. In current study, we hypothesized that a novel approach based on DL methods could aid in detecting patients with CAD using only a conventional 12-lead ECG.

## RESULTS

### Study population

A total of 17679 patients with cCTA data were screened between June 2010 and December 2020 to form the initial study cohort for analysis. With predetermined exclusion criteria, 2329 patients (13.2%) were excluded. Among the remaining 15350 patients, we further excluded 4812 patients for whom ECG data were unqualified. Finally, a total of 10538 patients with efficient ECG-cCTA pairs were included, with a mean age of 60.9 ± 11.6 years, 56.4% being male, and 4300 having significant CAD, with a CAD incidence of 40.8%. As expected, the ECGs of more than one-half of CAD patients (65.7%) were completely normal. Baseline characteristics of the included patients were presented in Table 1.

**Table 1:** Clinical characteristics of all enrolled patients.

	**Overall**	**CAD patients**	**Non-CAD patients**	***P*-value**
*n*	10538	4300 (40.8%)	6238 (59.2%)	
Age (years)	60.9 ± 11.6	64.9 ± 10.5	58.2 ± 11.5	<0.001
Sex				<0.001
Male	5943 (56.4%)	2799 (65.1%)	3144 (50.4%)	
Female	4595 (43.6%)	1501 (34.9%)	3094 (49.6%)	
Department				<0.001
Cardiology	4974 (47.2%)	3079 (71.6%)	1895 (30.4%)	
Outpatient	2729 (25.9%)	430 (10.0%)	2299 (36.8%)	
Cardiothoracic surgery	485 (4.6%)	176 (4.1%)	309 (5.0%)	
Examination center	337 (3.2%)	52 (1.2%)	285 (4.6%)	
Chinese medicine	316 (3.0%)	69 (1.6%)	247 (4.0%)	
Neurology	306 (2.9%)	99 (2.3%)	207 (3.3%)	
Endocrinology	285 (2.7%)	86 (2.0%)	199 (3.2%)	
Orthopaedic	116 (1.1%)	43 (1.0%)	73 (1.2%)	
Gastroenterology	116 (1.1%)	30 (0.7%)	86 (1.4%)	
Others	874 (8.3%)	236 (5.5%)	638 (10.2%)	
ECG				<0.001
Normal	7223 (68.5%)	2825 (65.7%)	4398 (70.5%)	
ST-segment changes	1415 (13.4%)	518 (12.0%)	897 (14.4%)	
T-wave changes	788 (7.5%)	354 (8.2%)	434 (7.0%)	
Atrial fibrillation	237 (2.2%)	112 (2.6%)	125 (2.0%)	
LBBB or RBBB	158 (1.5%)	64 (1.5%)	94 (1.5%)	
LV hypertrophy	124 (1.2%)	78 (1.8%)	46 (0.7%)	
Atrioventricular block	101 (1.0%)	62 (1.4%)	39 (0.6%)	
Others	492 (4.7%)	287 (6.7%)	205 (3.3%)	

Data presented as mean ± standard deviation or *n* (%). Abbreviations: CAD: coronary artery disease; LBBB: left bundle branch block; RBBB: right bundle branch block; LV: left ventricular. *P*-value was obtained by comparison of the CAD and Non-CAD patients.

**Figure 1:**
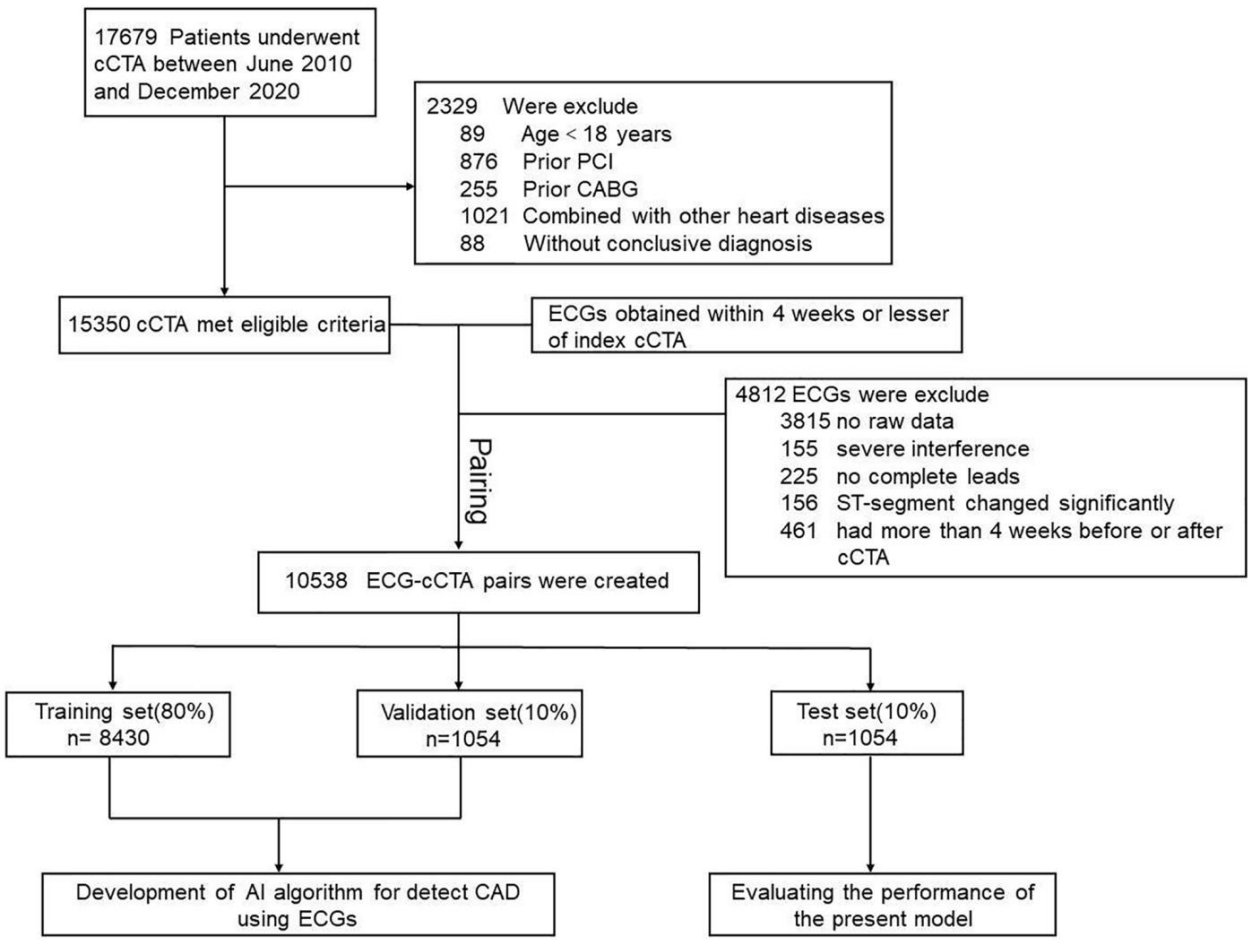
**Study flowchart.** Abbreviations: CAD: coronary artery disease; cCTA: coronary computed tomography angiography; ECG: electrocardiogram; CABG: coronary artery bypass grafting; PCI: percutaneous coronary intervention.

All enrolled data pairs were randomly assigned to one of the three datasets: a training dataset (80%, *n* = 8430), a validation dataset (10%, *n* = 1054), and a test dataset (10%, *n* = 1054). The flow chart in Figure 1 depicted the detailed screening and classification process for patients enrolled in this study and Table 2 showed the baseline characteristics of the three datasets.

**Table 2:** Clinical characteristics of the three datasets.

	**Training set**	**Validation set**	***P*-value^a^**	**Test set**	***P*-value^b^**
	**(*n* = 8430)**	**(*n* = 1054)**		**(*n* = 1054)**	
CAD			>0.05		<0.05
Yes	3414 (40.5%)	422 (40%)		464 (44%)	
No	5016 (59.5%)	632 (60%)		590 (56%)	
Sex			>0.05		<0.001
Male	4697 (55.7%)	590 (56%)		656 (62.2%)	
Female	3733 (44.3%)	464 (44%)		398 (37.8%)	
Age(years)	60.9 ± 11.7	60.8 ± 11.8	>0.05	62.8 ± 10.6	>0.05
ECG			<0.001		>0.05
Normal	6025 (71.5%)	588 (55.8%)		610 (57.8%)	
ST-segment changes	1053 (12.5%)	171 (16.2%)		191 (18.1)	
T-wave changes	618 (7.3%)	79 (7.5%)		91 (8.6%)	
Atrial fibrillation	179 (2.1%)	31 (2.9%)		27 (2.6%)	
LBBB or RBBB	119 (1.4%)	18 (1.7%)		21 (2.0%)	
LV hypertrophy	85 (1.0%)	20 (1.9%)		19 (1.8%)	
Atrioventricular block	70 (0.8%)	16 (1.5%)		15 (1.4%)	
Others	281 (3.3%)	131 (12.4%)		80 (7.6%)	

Abbreviations: LV: left ventricular; LBBB: left bundle branch block; RBBB: right bundle branch block. ^a^*P*-value was obtained by comparison of the Training set and Validation set. ^b^*P*-value was obtained by comparison of the Training set and Test set.

### Model performance

**Figure 2:**
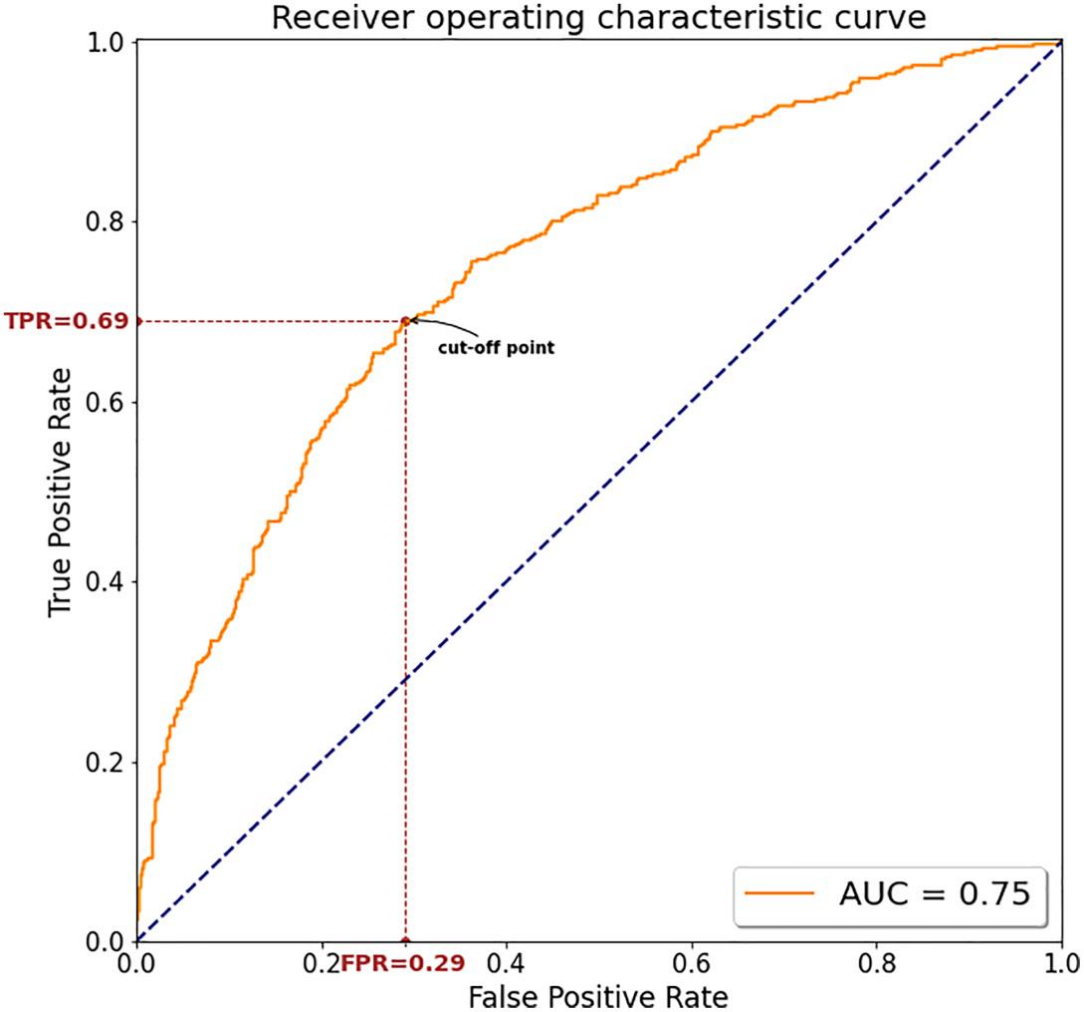
**The ROC curve for the model's screening performance on the test dataset using 8 leads.** The minimum distance between the ROC curve and the upper left corner was used to determine the optimal cutoff for best discrimination between CAD and non-CAD. Abbreviations: AUC: area under the curve; TPR: true positive rate; FPR: False Positive Rate.

**Figure 3:**
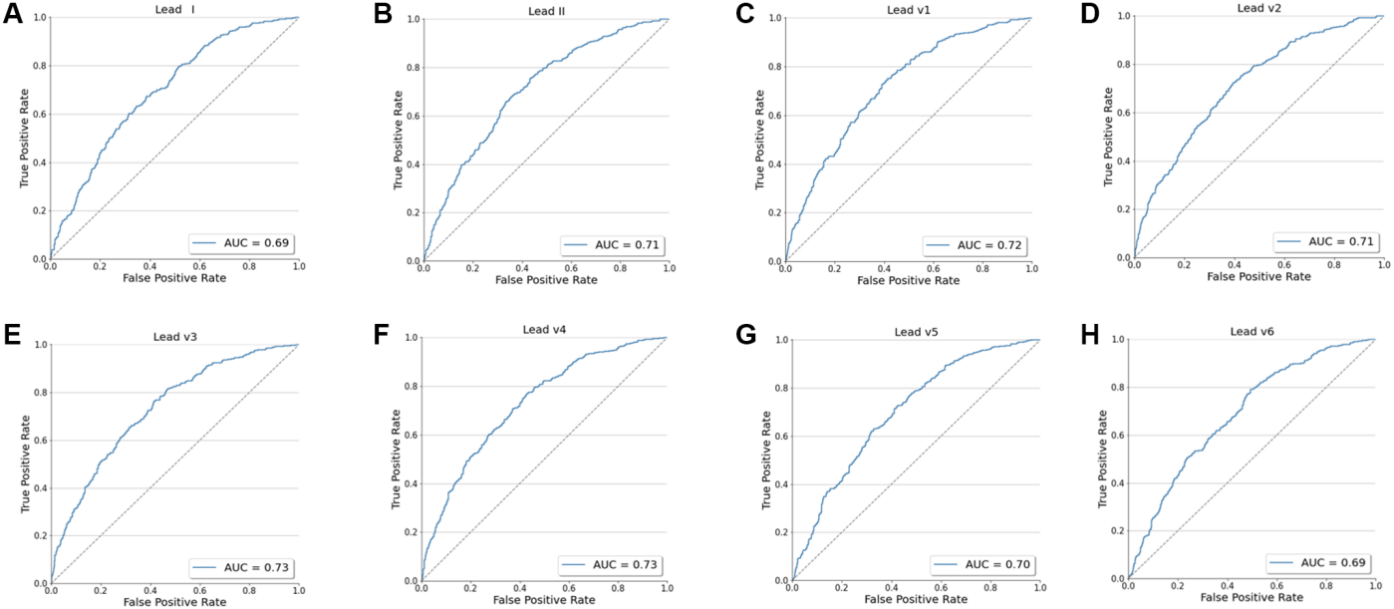
**The ROC curve for the model's screening performance on the test dataset using every single lead (I, II, V1-V6).** (**A**) The ROC curve for I lead. (**B**) The ROC curve for II lead. (**C**) The ROC curve for V1 lead. (**D**) The ROC curve for V2 lead. (**E**) The ROC curve for V3 lead. (**F**) The ROC curve for V4 lead. (**G**) The ROC curve for V5 lead. (**H**) The ROC curve for V6 lead.

Table 3 illustrated the performance metrics on the test dataset. The accuracy of the CNN model using 8 leads in detecting CAD patients was 70.0% (95% CI, 69.5% to 70.1%), with a sensitivity of 68.7% (95% CI, 68.1% to 68.9%), specificity of 70.9% (95% CI, 70.3% to 71.0%), PPV of 61.2% (95% CI, 60.6% to 61.2%), and NPV of 77.2% (95% CI, 76.8% to 77.3%). Table 4 illustrated the model’s performance to identify CAD using every single lead of the ECG. Among them, the V3 and V4 lead had the same and largest AUC (0.73). Table 5 summarized the confusion matrix of CNN model using single lead and cCTA for classification of CAD. In addition, we evaluated the ability of these two models (8 leads and single lead) to screen patients with CAD separately using ROC and calculate the area under the ROC curve (AUC) (Figures 2 and 3).

**Table 3:** Confusion matrix for classification of CAD, the model (8 leads) vs. cCTA.

		**Predicted**						
		**CAD**	**Non-CAD**	**Acc**	**PPV**	**NPV**	**Sen**	**Spec**
Original	CAD	290	132	0.7	61.20%	77.20%	68.70%	70.90%
	Non-CAD	184	448	(0.69–0.70)	(60.0%–61.2%)	(76.8%–77.3%)	(68.1%–68.9%)	(70.3%–71.0%)

The model performance in predicting CAD was assessed in the test group. Abbreviations: CAD: coronary artery disease; cCTA: coronary computed tomography angiography; Acc: accuracy: PPV: positive predictive value; NPV: negative predictive value; Sen: sensitivity; Spec: specificity.

**Table 4:** Test data set performance for CAD from single lead of the ECG.

**Lead**	**PPV**	**NPV**	**Sen**	**Spec**	**AUC**
I	82.60%	51.00%	40.00%	88.10%	0.69
II	56.10%	74.50%	66.60%	65.20%	0.71
V1	64.10%	67.20%	37.20%	86.10%	0.72
V2	56.80%	72.10%	59.50%	69.80%	0.71
V3	64.40%	69.40%	41.90%	84.50%	0.73
V4	63.80%	68.50%	42.20%	84.00%	0.73
V5	73.90%	68.40%	37.00%	91.30%	0.70
V6	51.30%	72.60%	67.80%	57.00%	0.69

The model performance in predicting CAD was assessed in the test group. Abbreviations: CAD: coronary artery disease; cCTA: coronary computed tomography angiography; PPV: positive predictive value; NPV: negative predictive value; Sen: sensitivity; Spec: specificity; AUC: area under the receiver operating characteristic curve.

**Table 5:** Confusion matrix for classification of CAD, the model (single lead) vs. cCTA.

**Lead**	**Test data set (*n* = 1054) No. (%)**				
	**True positive**	**False positive**	**True negative**	**False negative**	**Accuracy**
I	247 (23.4)	52 (4.9)	385 (36.5)	370 (35.2)	632 (60.0)
II	281 (26.6)	220 (20.9)	412 (39.1)	141 (13.4)	693 (65.7)
V1	157 (14.9)	88 (8.3)	544 (51.6)	265 (25.2)	701 (66.5)
V2	251 (23.8)	191 (18.1)	441 (41.8)	171 (16.3)	692 (65.7)
V3	177 (16.8)	98 (9.3)	534 (50.7)	245 (23.2)	711 (67.5)
V4	178 (16.9)	101 (9.6)	531 (50.4)	244 (23.1)	709 (67.3)
V5	94 (8.9)	55 (5.2)	577 (54.7)	328 (31.2)	671 (63.7)
V6	286 (27.1)	272 (25.8)	360 (34.2)	136 (12.9)	646 (61.3)

The model performance in predicting CAD was assessed in the test group. Abbreviations: CAD: coronary artery disease; cCTA: coronary computed tomography angiography.

## DISCUSSION

In this study, we developed a deep learning-based model to detect CAD using only ECG data. In the test dataset, our model using 8 leads performed moderately in detecting CAD, with an AUC of 0.75 (95% CI, 0.73 to 0.78), accuracy of 70.0% (95% CI, 69.5% to 70.1%), sensitivity of 68.7% (95% CI, 68.1% to 68.9%), specificity of 70.9% (95% CI, 70.3% to 71.0%), PPV of 61.2% (95% CI, 60.6% to 61.2%), and NPV of 77.2% (95% CI, 76.8% to 77.3%). In order to further explore the ability of the AI model to identify CAD and consider the practical application of the model, we also trained the CNN network on 8 single-lead (lead I, II, V1-V6) and also evaluated the AI model’s ability to identify CAD in the test dataset using every single-lead. However, we found that the AUC of the model using single lead to identify CAD is generally low. Although the model of V3 and V4 lead achieved an AUC of 0.73, the AUC was 0.02 higher using 8 leads of the ECG and we believe that this difference is mainly due to the following reasons: first, when CAD is in its early stages, the ECG may only show minimal alterations even normal, in other words, there are no obvious characteristics of ECG changes in the early stage of CAD. Therefore, the model may not be able to extract valuable features from single-lead ECG only. On the contrary, we believe that 8 leads can provide a richer dimension and more information, and the model can learn more valuable features, so as to produce a better recognition result. In addition, deep learning often requires a large amount of data. After increasing the amount of data, the model using single lead may extract more valuable features, and may also achieve a better recognition ability. Nonetheless, these findings demonstrated possibility and feasibility of developing a well-trained CNN model to detect CAD patients using only a standard 12-lead ECG.

CAD is a common disease that continue to be the leading cause of mortality and morbidity worldwide, imposing enormous health and economic burdens. Delayed diagnosis and treatment of CAD can have significant consequences for patients, such as myocardial infarction (MI) and ischemia-induced congestive heart failure (CHF), which is linked to a lower quality of life [24]. As a result, for CAD patients, timely diagnosis and early intervention are critical factors in improving prognosis.

Many cardiac imaging technologies, such as transthoracic echocardiography (TTE), magnetic resonance imaging (MRI) and myocardial perfusion imaging, have been widely used to detect cardiac function or structure injury. These methods, however, are time consuming and expensive, making them unsuitable for CAD screening. Coronary angiography (CAG) has long been regarded as the ‘gold standard’ for detecting CAD. However, only patients with typical clinical signs and symptoms or high risks for CAD were ever referred to CAG for further evaluation and treatment [25]. As a result, in the current study we defined CAD based on cCTA to reduce the selection bias.

ECG is a fundamental tool in the everyday practice of clinical medicine due to its wide availability and low cost and has frequently been used to non-invasively detect and diagnose cardiac arrhythmia and acute coronary syndromes. When CAD is in its early stages, the ECG may only show minimal alterations and are difficult to detect by the naked eye. We hypothesized that AI could assist us in diagnosing asymptomatic CAD by detecting minor alterations and delivering relevant information.

To test our idea, we created a novel approach based on deep learning algorithm as a reliable CAD screening tool. DL is a collection of new techniques. It has shown advantages of automatically discovering best representations needed for classification task from raw data without handcrafted feature engineering. The greatest advantage of DL is its ability to process various complex data, such as time-series data, 2D data and images. In the CNN algorithm, we used the raw ECG data (numerical matrix, 4000 × 8) rather than the ECG image itself to ensure that the complete information of the ECG raw signal was fully utilized. Although more computational power was needed to process and use the raw signal for the CNN in the model’s training process, we could use the features of ECG itself which were extracted by the CNN model over human bias. Similar to our use of ECG raw data for the diagnosis of CAD, a research from the Mayo Clinic [23] showed that a CNN model based on DL technology was able to detect patients with asymptomatic left ventricular dysfunction (ALVD), defined as ejection fraction ≤35% by echocardiography, using the ECG data alone and demonstrated its feasibility. Indeed, the ECG is an excellent substrate for AI applications, which yields reproducible and large amounts of raw data, such as portable wearable ECG device. Importantly, such large amounts of ECG data are relatively easy to store and converted to a digital format [17].

In fact, the unprecedented progress in DL technology have enabled computers to make an accurate automatic interpretation and diagnose of ECG in the early stage of the disease. Galloway et al. [26] developed an AI–ECG model to screen hyperkalemia in patients with chronic kidney disease. At an appropriate cut-off point, the model yielded values for the sensitivity and specificity of 90%, 89% respectively in a multicenter, external validation. Diagnosis delay is a clinical conundrum in acute ST-segment elevated myocardial infarction (STEMI). Base on ECG alone, Zhao et al. [22] created an AI-based STEMI autodiagnosis algorithm to identify STEMI, which achieved an AUC of 0.9954 with high accuracy, specificity, and sensitivity of 99.01%, 99.20%, 96.75% respectively, in the external evaluation. In our study, considering that the ECG showed no any specific changes and may only occur minor alterations in the early stages of CAD. Therefore, in order to improve diagnostic accuracy, we embedded the SE module into the original ResNet-50 network structure, which considered the interdependence of the model channels and could reinforce the extracted important features. To our knowledge, such model construction method is rarely seen in the study of its kind. Although compared with these similar studies, our model only yielded moderate performance (AUC 0.75). We thought it may be related to the following reasons. First, whether hyperkalemia or STEMI, the ECG features of this kind of disease are often more obvious, and the model is easier to extract such abnormal features. Second, as shown in Table 1, the ECGs we enrolled were from patients with various diseases in different departments. The underlying disease may also cause ECG changes, which might interfere with the model extracting ECG features related to CAD. In addition, non-specific changes in ECG such as ST-segment changes, flat T, T wave inversion, and so forth may affect or conceal the ECG features related to CAD, thereby affecting the effectiveness of the model to screen for CAD. Nevertheless, we still believe our research holds significant implications, because it is almost impossible for clinicians to judge whether a patient has potential CAD based only on the ECG.

The traditional automatic diagnosis of ECG mainly relies on ML algorithms and is mainly used in the diagnosis of acute coronary syndrome and arrhythmia [18]. However, the traditional ML algorithm mainly uses artificially extracted time or ECG morphological features as input, such as QT interval, RR interval or P wave amplitude, ST segment elevation et al. Advanced, it is not only time-consuming and labor-intensive, but also the selection of features is very dependent on the knowledge and experience of researchers [27]. Different from the traditional ML algorithm, the DL algorithm can directly process the ECG original signal, realize the layer-by-layer feature extraction of the input signal, get rid of the dependence and restraint on the prior knowledge and manual feature extraction, and thus realize a new approach that unifies data preprocessing, feature extraction and classification into one framework. In other words, the CNN structure can accomplish all these steps “end-to-end” without requiring class-specific feature extraction [28]. Currently, DL is mostly used to identify myocardial infarction [22, 29] or arrhythmia [30] in the automatic diagnosis of ECG, because the ECG characteristics of these types of heart diseases are more obvious, and the feature identification is relatively easy, and the results are often better. However, what we’ve done is to identify patients with coronary artery disease from the ECG that is basically normal or has no obvious specific changes. To our knowledge, our study showed for the first time to explore the application of a deep learning–based algorithm for detecting CAD using only ECG. Our model yielded moderate values (AUC 0.75, sensitivity 68.7%, specificity 70.9%) in the test dataset, which showed a comparable performance to the traditional Diamond–Forrester model [31] and the widely available CAD consortium model [32]. The Diamond–Forrester model provided an easy way for determining the pre-test CAD probability by using simple demographic data and clinical factors which included sex, age and type of chest pain [31, 33]. However, it has been shown that this model overestimates CAD risk in more contemporary populations because of the obsolescence of model development population [34]. Although other updated models such as CAD consortium model included more clinical factors and have improved performance and versatility, the multi-dimensional variables contributed to the complexity of the model and difficulty in clinical application [32, 35–37].

It is well known that CAD is related to many factors, such as sex, age, weight, body mass index, smoking status, systolic blood pressure, and so forth. Zheng et al. [38] developed a CNN model to detect CAD patients based on facial photos, which achieved moderate performance of AUC 0.730, sensitivity of 80.0% and specificity 54.0% in an independent test dataset. However, they fund it did not further improve the model’s performance when additional clinical variables were added. That is, it is easy to use this model only based on facial photos without additional demographic data or clinical variables. However, as stated by the author, privacy protection and data security were major obstacle to the application of this technology. Similarly, in the present study, our model did not use any demographic data or clinical variables but input ECG raw data only for model training, validation, testing mainly because the following reasons. First, to avoid selection bias, we included patients from all departments of the hospital and nearly 30% of the enrolled patients were from outpatient and physical examination center. For these patients, we could not get access to the clinical variables from the healthcare system of our hospital. Second, too many variables will increase the complexity of the model, which is not conducive to the practical application of the model. In addition, we believe that because the research of DL technology in ECG is still in the early stage, too many additional variables may conceal the actual screening ability of DL model based on ECG. Therefore, we are committed to improving the diagnosis accuracy of CAD based on the ECG raw data alone through DL technology. Fortunately, our model also obtained moderate performance for detecting CAD (AUC 0.75, sensitivity 68.7%, specificity 70.9%).

This study showed the possibility and feasibility of a deep learning-based algorithm for CAD screening only based on ECG. While this area of research is still in relative infancy, there is reason to be optimistic about the applications of such technology in the future, that is, the AI-ECG could be implemented into early or asymptomatic CAD screening as an effective, low-cost, and non-invasive assay. Even though the model training process were highly reliant on significant computing power and time, however, once the model was complete, only a smaller amount of computational power is needed to analyze and interpret the input ECG. In future clinic, for patients with suspected asymptomatic CAD, the computers using our algorithm can make prediction on ECG inputs and the prediction result will be sent back and displayed to the clinicians. Then, clinicians may interpret the result combining with patients’ general conditions and clinical symptoms or signs. According to the comprehensive results, physicians can help patients guide some general recommendations or further diagnostic tests. However, it is clear that we still have a long way to go before the actual clinical applications of this tool, which requires the joint effort of scientific researchers and clinicians around the globe.

Some limitations of our study should be noted. First, since the present study was based at only one hospital, the lack of external validation may result less accuracy compared with the performance in internal validation. So additional validation in patients from other hospitals even other countries and races would be necessary to examine model’s performance. However, it does not affect our main purpose of evaluating the feasibility of detecting CAD using ECG alone. In the near future, we will first plan to launch a multi-center validation of this AI algorithm in our city. Second, it is currently unknown how deep learning algorithm contribute to decision process. The lack of transparency inherent to CNN has been linked to a “black box”, which makes it difficult for clinicians to understand how exactly the AI arrives at a solution. Third, in our study, the relatively low specificity (70.9%) and up to 29.1% false-positive rate may place additional confusion and anxiety on patients. Thus, identifying a higher risk population that might benefit most from the algorithm based on basic clinical data should be explored, which is also our future study direction.

## CONCLUSION

In this study, we developed a model based on deep learning algorithm, which brought up feasibility and possibility of predicting the presence or absence of CAD. The AI-ECG could be as a relatively low cost, noninvasive and efficient screening tool for CAD only using routine 12-lead ECG. Additional prospective studies from multicenter will be urgently needed to accurately access the screening ability of the model and determine whether the AI-ECG can relieve some economic burden on CAD patients as well as the health care system.

## MATERIALS AND METHODS

### Participants

Patients who were evaluated for suspected CAD and underwent clinically indicated cCTA were included in the study. This study excluded: (i) minors (aged <18 years); (ii) prior coronary artery bypass grafting (CABG) surgery; (iii) prior percutaneous coronary intervention (PCI); (iv) history of myocardial infarction or clinically significant cardiac disease (e.g., congenital heart disease, rheumatic heart disease, or aortic disease); (v) ECG with severe interference or missing leads; (vi) ECG suggested myocardial ischemia or ST-segment changed significantly (ST-segment elevation/depression >0.1 mV in at least 2 contiguous ECG leads) and (vii) ECG or cCTA information was missing (e.g., without ECG raw data or conclusive cCTA diagnosis) (Figure 1).

### Data collection and pairing

We respectively collected ECG and cCTA data from the cardiac electrophysiology laboratory and radiology department of Sun Yat-sen Memorial Hospital, Sun Yat-sen University from 2010 to 2020. ECG signals were obtained at rest in supine position using a 1250-P ECG machine (Nihon Kohden) with a sampling frequency of 500 Hz, and all the ECGs were formatted as raw data for further analysis. All enrolled patients were divided into two groups based on presence or absence of CAD, with CAD defined as the presence of no less than 50% stenosis in at least one major coronary artery (left main artery, left anterior descending coronary artery, left circumflex artery or right coronary artery) based on cCTA [31, 39]. The cCTA results of each patient were reviewed by two qualified radiologists to determine the presence and severity of coronary stenosis. If the first 2 radiologists disagreed, a third senior cardiologist was consulted and a final decision was reached. Each ECG for each included case was matched to one cCTA based on the hospitalization or outpatient ID.

Then the standard 10-second, 12-lead ECG and cCTA performed at 4 weekly intervals or lesser from each adult patient were paired. The ECG raw data were used as inputs to train, validate and test the CNN model for each paired ECG-cCTA, with cCTA labels indicating whether or not a case had CAD.

### Processing of ECG data

**Figure 4:**
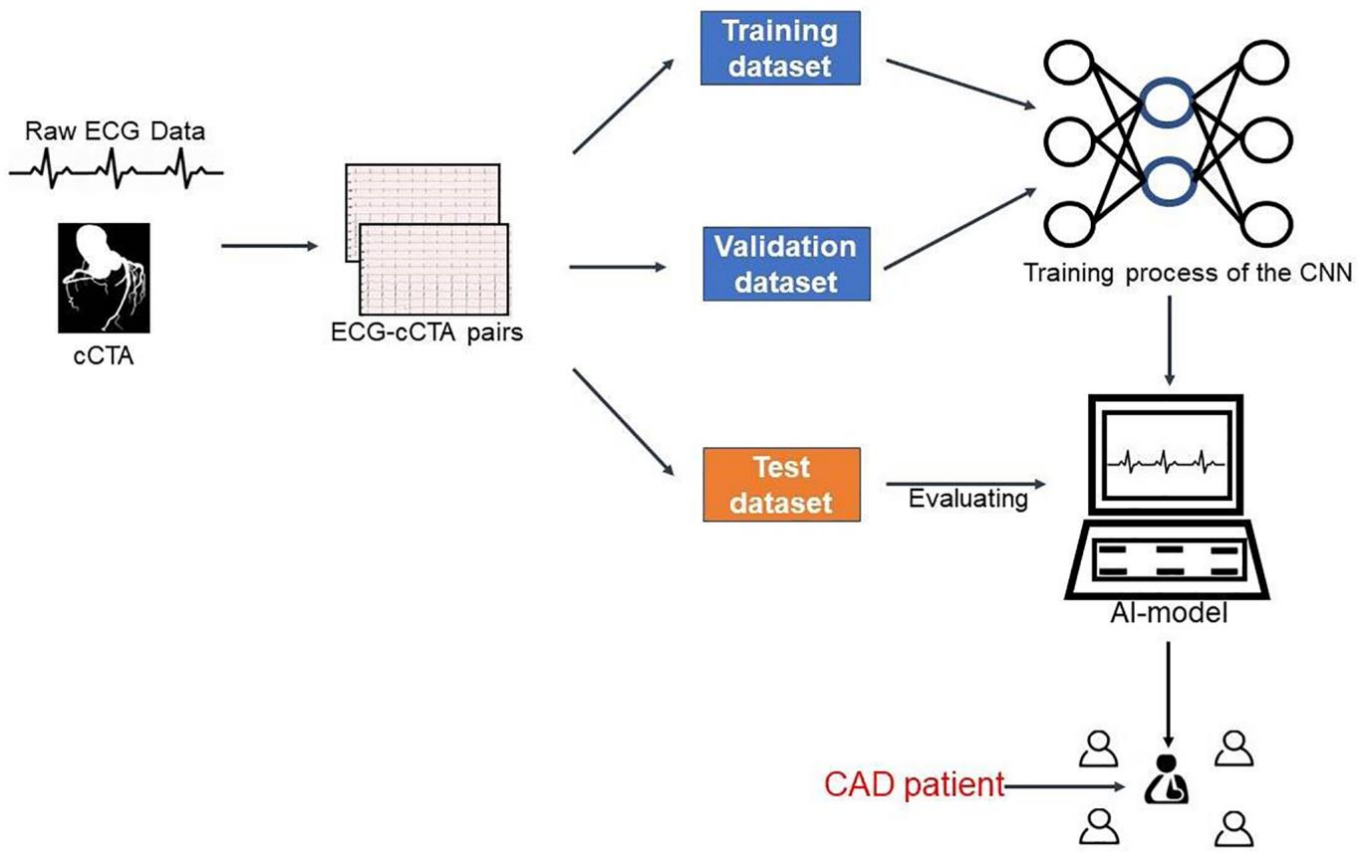
**Integrated process for developing and testing the AI model.** Abbreviations: cCTA: coronary computed tomography angiography; ECG: electrocardiogram; AI: artificial intelligence; CAD: coronary artery disease; CNN: convolutional neural network.

Each raw digital ECG data consisted of a 5000 × 12-matrix (500 Hz data for 10 seconds in 12 leads). This study included eight independent ECG leads of I, II and V1-V6, since the four augmented leads (avR, avL, avF, III) are created as a linear function of leads I and II and do not contain incremental information [15]. As a result, a 5000 × 12-matrix was reduced to a 5000 × 8-matrix. Furthermore, in order to further explore the ability of the AI model to identify CAD, we trained the CNN network on 8 single-lead (lead I, II, V1-V6) and also evaluated the AI model’s ability to identify CAD in the test dataset using every single-lead. And according to the research reported, some leads (lead I, II, V2) have been used to enable patient self-monitoring [40]. The whole dataset was randomly divided into training/validation/test sets with a proportion of 8:1:1. We built the model using the training and validation datasets, and then tested it on the test dataset. Figure 4 depicted the overall model creation process.

### Overview of the deep-learning model

**Figure 5:**
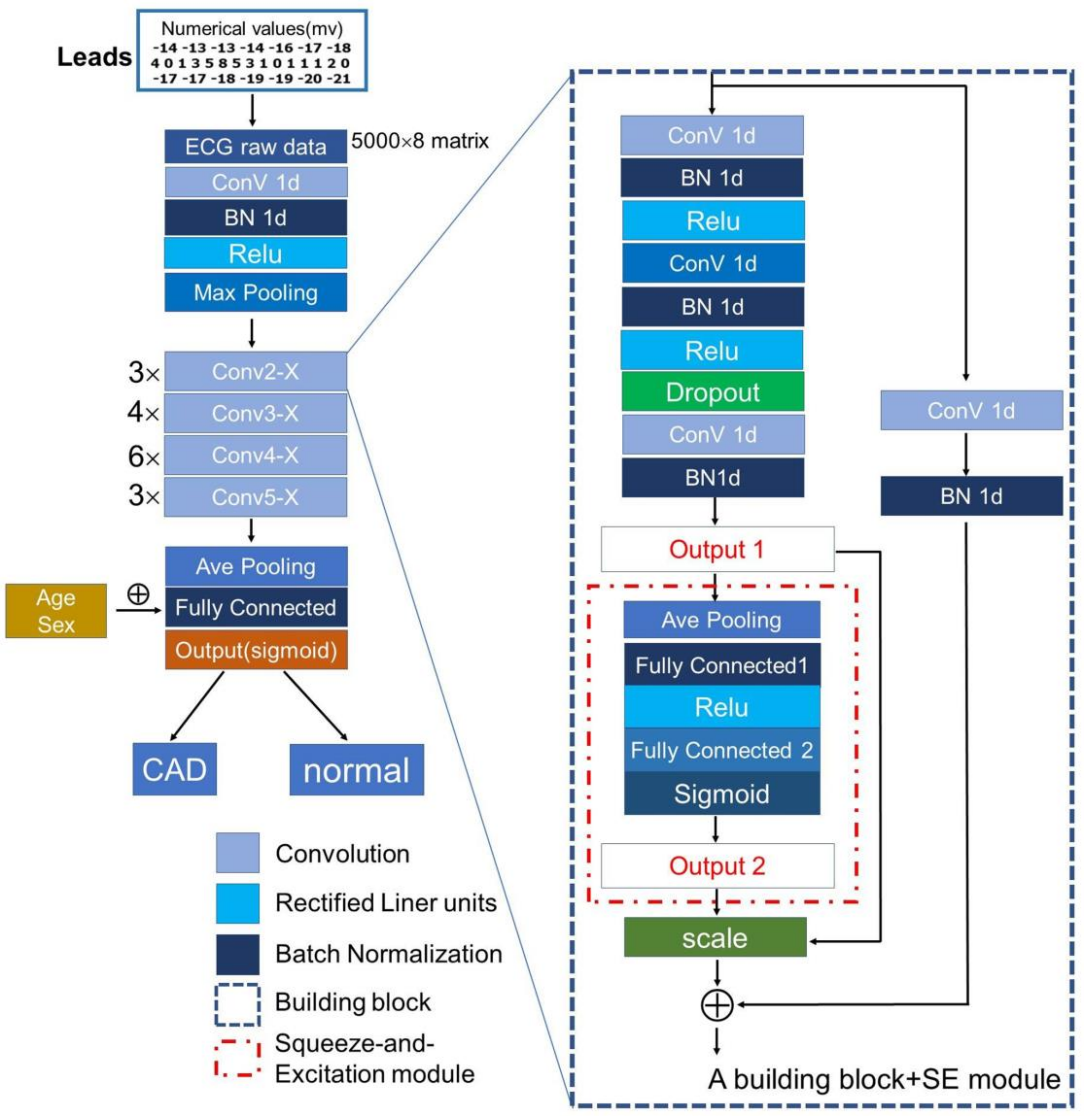
**The architecture of the proposed CNN model.** We used ResNet-50 uniting a mixed “attention” module (the Squeeze-and-Excitation module) as backbone architecture to extract useful features from the input ECG raw data. Abbreviations: ConV: Convolution; BN: Batch Normalization; Relu: Rectified Linear Units.

Deep learning is a method which employs many hidden layers of neurons to learn arbitrarily complex data features at multiple levels of abstraction. It is ideal for classification task of complex medical data, such as ECGs. A typical CNN is constructed using convolutional layers, pooling layers and fully-connected layers [41], with convolutions layers extracting useful features from the input dataset, which in our current study is ECG signals. The pooling layers are used to reduce the dimensions of the feature maps while retaining useful information. Finally, the fully-connected layers connect all of the previous layer’s inputs, followed by an output layer. In our study, we used a CNN with ResNet-50 backbone architecture and a Squeeze-and-Excitation (SE) module as our classifier model. We created the SeResNet-50 network by embedding the SE module into the original ResNet-50 network structure, with a slightly increased computational cost and markedly enhanced performance. The SE module’s main consideration was the interdependence of the model channels. Graphical representation of the network’s general architecture was depicted in Figure 5. A deep learning framework called PyTorch (version1.7.1, Facebook) was used for training and evaluation of our network, which was implemented using the Ubuntu operating system through the use of Numpy, Pandas and scikit-learn libraries.

We used a backpropagation algorithm with an 85-batch size to update model parameters after calculating the binary cross-entropy loss function between predicted and real values. This deep learning model was optimized using Adam optimizer with the following parameters: weight decay (L2 regularization) = 2 × 10^−5^, learning rate = 3 × 10^−3^ with poly learning rate scheduler. These parameters can help with overfitting issues, achieve fast data convergence, and adjust the learning speed.

### Model evaluation

Our study’s primary goal was to see if the AI model could tell the difference between patients with and without CAD just by looking at their ECGs. The model calculated the likelihood of CAD on the scale of 0 (non-CAD) to 1 (CAD). The model’s performance characteristics, such as sensitivity, specificity, PPV, NPV and diagnostic accuracy were calculated. Because the output was continuous, the final classification decision was made by choosing a threshold and testing whether the output was below or above it. The receiver operating curve (ROC) displayed all possible thresholds with their associated sensitivity and specificity, and the area under the receiver operating curve (AUC) was calculated.

### Statistical analysis

The baseline characteristics of the CAD and control groups were compared. Continuous variables that exhibited a normal distribution were shown as mean ± standard deviation (SD) and tested using the unpaired Student’s test. Categorical variables were represented as percentages and analyzed using Chi-square tests or Fisher’s exact tests. Two-sided *P* values of less than 0.05 were considered statistically significant. Measures of diagnostic performance were summarized using 2-sided 95% confidence intervals (CIs). The optimal cut-off point for discriminating between CAD and non-CAD was determined using the minimum distance between the ROC curve and the upper left corner or the Youden index (specificity + sensitivty-1). All routine statistical analyses were performed using R, version 3.4.2 (R Foundation).
